# Exploring Therapeutic Potential of Bi-Qi Capsules in Treatment of Gout by Discovering Crucial Drug Targets

**DOI:** 10.3390/ph18050618

**Published:** 2025-04-24

**Authors:** Jing Xie, Yu Zhang, Rong Ren, Ruizhen Bu, Liying Chen, Juezhuo Hou, Dandan Shang, Yadong Liu, Dan Wang, Tao Wang, Hong Zhou

**Affiliations:** 1 State Key Laboratory of Bioactive Substance and Function of Natural Medicines, Institute of Materia Medica, Chinese Academy of Medical Sciences and Peking Union Medical College, Beijing 100050, China; xiejingdrt@163.com (J.X.); nkwangdan@163.com (D.W.); 2 Tianjin Pharmaceutical Da Ren Tang Group Co., Ltd., 17 Baidi Road, Nankai District, Tianjin 300193, China; zhangyu@jydrt.com.cn (Y.Z.); renrong@tjph.cn (R.R.); houjuezhuo@zx-innova.com (J.H.); shangdandan@zx-innova.com (D.S.); liuyadong@zx-innova.com (Y.L.); 3 Tianjin Key Laboratory of Quality Control in Chinese Medicine, 21 10th Street, Binhai New Area, Tianjin 300457, China; chenliying@ymj.cn; 4 Tianjin Darentang Jingwanhong Pharmaceutical Co., Ltd., 20 Daming Road, Xiqing District, Tianjin 300112, China; buruizhen@126.com; 5 Tianjin Pharmaceutical Da Ren Tang Group Co., Ltd., Traditional Chinese Pharmacy Research Institute, 21 10th Street, Binhai New Area, Tianjin 300457, China; 6 Department of Pharmacology, Academy of Traditional Chinese Medicine, Tianjin University of Traditional Chinese Medicine, 10 Poyang Lake Road, Jinghai District, Tianjin 301617, China

**Keywords:** gout, Bi-Qi capsule, network pharmacology, Mendelian randomization, traditional Chinese medicine

## Abstract

**Objectives**: This research aims to explore the therapeutic potential of Bi-Qi capsules in the treatment of gout by identifying crucial drug targets through a multidimensional data analysis strategy. **Methods**: Bi-Qi capsule drug targets and differentially expressed genes (DEGs) of gout were derived from public databases, such as Swiss Target Prediction, STITCH, and the GEO database. Subsequently, the overlapped targets were analyzed to elucidate the potential therapeutic mechanism and to identify candidate targets of Bi-Qi capsules against gout. Next, Mendelian randomization (MR) analysis was employed to screen and explore the causal relationship between candidate targets and gout. Finally, single-cell RNA sequencing (scRNA-seq), gene set enrichment analysis (GSEA), transcription factor and ceRNA regulatory networks, and molecular docking were performed to validate the role of the crucial targets of Bi-Qi capsules in the treatment of gout. **Results**: A total of 46 candidate targets were identified, in which KCNA5, PTGS2, and TNF exhibited significant causal relationships with gout (*p* < 0.05) and were regarded as the crucial targets. Through scRNA-seq and gene labeling, crucial targets were found to be expressed in eighteen cell clusters and eight cell types, which are closely associated with carbohydrate metabolism, nerve conduction, and the innate immunity process. Bi-Qi capsule active compounds such as tanshinone IIA, strychnine, tanshinaldehyde, cryptotanshinone, tumulosic acid, and glycyrrhetic acid exhibit a better binding ability to crucial targets. **Conclusions**: The results not only elucidate the anti-gout mechanism of Bi-Qi capsules but also provide an insight into multi-target natural medication for metabolic disease treatment, which contributes to guiding the clinical application of Bi-Qi capsules in the future.

## 1. Introduction

Gout, as the most common form of chronic inflammatory arthritis, is triggered by the deposition of monosodium urate crystals, which are the end product of human purine metabolism, into and around the articular tissues, soft tissues, and bones [1,2]. It is characterized by acute attacks of severe pain, swelling, redness, and heat in one or more joints and may even develop into chronic gouty arthropathy, uric acid nephrolithiasis, or chronic nephropathy. The risk factors for gout include hyperuricemia (HUA), genetic susceptibility, gender, age, diet, lifestyle, medication, chronic diseases, and others [3]. As early as 2017, it was estimated that approximately 41.2 million adults worldwide are suffering from gout, and the number of people affected by gout exceeds more than twice that affected by rheumatoid arthritis [4,5]. Currently, the epidemiological data on gout show a substantial and growing trend all over the world [6]. In 2020, it was reported that 55.8 million adults were affected by gout with an age-standardized prevalence of 659.3 per 100,000, which is a 22.5% increase compared with 1990. Among them, the incidence rate in men is approximately 3.26 times higher than that in women. Considering population growth, the total number of prevalent gout cases could possibly reach 95.8 million by 2050 [7]. What is most worrisome is that gout gives rise to various complications easily, such as ischemic heart disease, myocardial infarction, hypertension, hyperlipidemia, chronic kidney disease, diabetes, metabolic syndrome, and dementia, which can lead to the increased mortality of patients [8,9,10,11]. The modern clinical treatment of gout mainly relies on pharmacotherapy, with nonsteroidal anti-inflammatory drugs (NSAIDs), colchicine, and systemic glucocorticoids serving as first-line treatments for acute gout attacks. Meanwhile, xanthine oxidase inhibitors, uricosuric agents, and urate oxidase are employed for urate-lowering therapy. However, they fail to absolutely eradicate the underlying causes of gout, and there exist notable limitations in terms of duration of use and safety concerns [12]. Given these limitations, there is an urgent need to develop novel anti-gout agents and therapeutic strategies to enhance disease prognosis by selectively targeting specific pathways involved in gout pathogenesis.

Bi-Qi capsules are a Chinese traditional patent medicine approved by the Chinese Food and Drug Administration (NMPA approval number: Z10910026) for treating bone disease, such as rheumatoid arthritis, cervical spondylosis, scapulohumeral periarthritis, and knee osteoarthritis [13,14,15,16]. Bi-Qi capsules were manufactured by the Tianjin Darentang Jingwanhong Pharmaceutical Co., Ltd., located in Tianjin, China. They consist of ten Chinese herbs, including Strychnosnux-vomica (Maqianzi), Pheretima aspergillum (Dilong), Radix Salviae miltiorrhizae (Dan Shen), Radix Codonopsis pilosula (Dang Shen), Poriacocos (Fuling), Panaxnotoginseng (Sanqi), Ligusticumwallichii (Chuanxiong), Rhizoma Atractylodis macrocephalae (Bai Zhu), Radix Achyranthis bidentata (Huai Niu Xi), and Radix glycyrrhizae (Gan Cao). Our previous research has preliminarily elucidated the pharmacological effects and mechanisms of Bi-Qi capsules in acute gouty arthritis; they are able to reduce serum uric acid levels, enhance uric acid clearance rates, and ameliorate renal injury, ankle joint swelling, and synovial injury by controlling the expression of adenosine deaminase, organic anion transporter 1, and glucose transporter 9, inhibiting the NLRP3 pathway and TLR4/NLRP3 pathway, reducing the levels of inflammatory factors [17]. However, the pharmacological effects and mechanisms of Bi-Qi capsules in treating gout have still not fully been explored.

**Figure 1:**
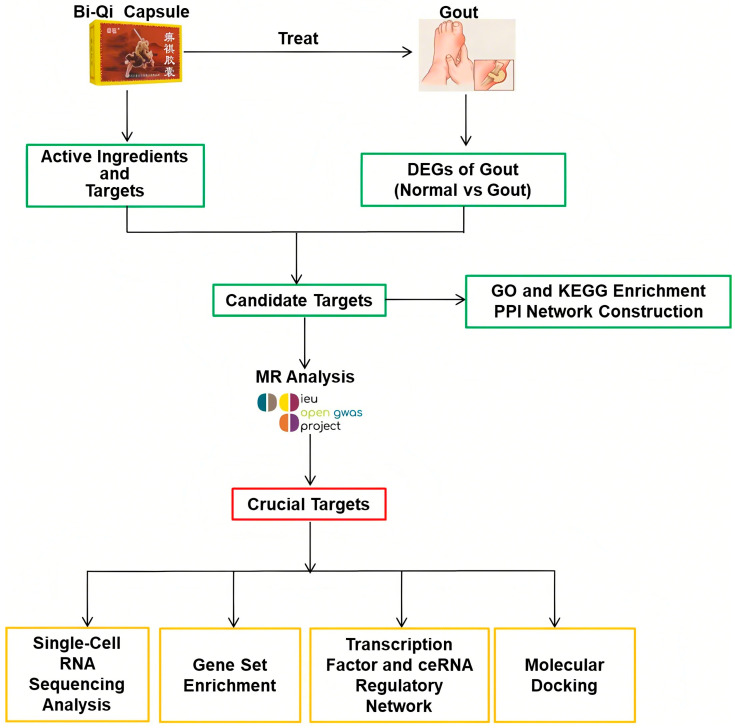
Study design in this research process.

Mendelian randomization (MR), also known as natural randomized controlled trials, provides a solution to the limitations of observational and interventional studies by mitigating the impact of confounding factors. The method employs genetic variation as an instrumental variable and constructs a model of “genetic variation–risk factors–disease outcomes” to probe into the potential causal relationships between various factors (disease, environment, society, behavior, psychology, etc.) and diseases [18,19]. At present, there is a growing trend of utilizing MR studies to identify drug targets by elucidating the relationships between drug target genes and diseases, which may disclose potential therapeutic mechanisms and crucial targets for disease management [20,21,22,23]. In this research, a multidimensional data analysis strategy integrating conventional bioinformatics methods with MR studies was used to explore the therapeutic potential of Bi Qi capsules in the treatment of gout (Figure 1).

## 2. Results

### 2.1. DEG Identification and Biological Function Analysis

**Figure 2:**
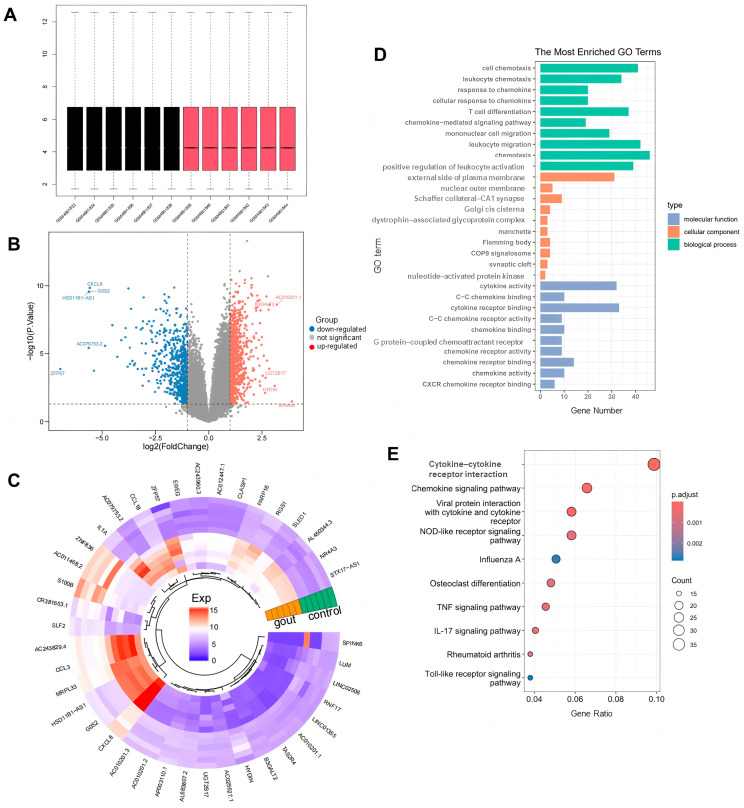
Screening differentially expressed genes (DEGs) between the normal group and gout group. (**A**) Uniformity test of gene expression distribution in each sample. Black is control group, red is gout group. (**B**) The volcano plot and (**C**) heatmap for the expression patterns of the DEGs. (**D**) Gene ontology (GO) functional enrichment analysis of DEGs. (**E**) Kyoto encyclopedia of genes and genomes (KEGG) functional enrichment analysis of DEGs.

In the GSE160170 dataset, a total of 1669 DEGs were identified in the normal and gout groups, including 941 up-regulated genes and 728 down-regulated genes, and the uniformity of gene expression distribution in each sample was good, as shown in Figure 2A. Volcano plots were established to describe the change trend of DEGs in a visualized manner, from which 20 genes with the largest differences in up-regulation and down-regulation were plotted on a circular heatmap (Figure 2B,C). GO and KEGG enrichment analyses mapped the prospective biological roles of DEGs. GO enrichment analysis revealed that DEGs largely referred to 30 related biological processes, cellular components, and molecular functions, such as chemotaxis, leukocyte migration and positive regulation, cytokine activity, T-cell differentiation, cytokine receptor binding, and other key biological phenomena (Figure 2D). KEGG enrichment analysis highlighted the top 10 signaling pathways, including cytokine receptor interactions, the chemokine signaling pathway, viral protein interactions with cytochrome and cytochrome receptors, the NOD-like receptor signaling pathway, and the TNF and IL-17 signaling pathways (Figure 2E).

### 2.2. Candidate Gene Identification and Biological Function Analysis

**Figure 3:**
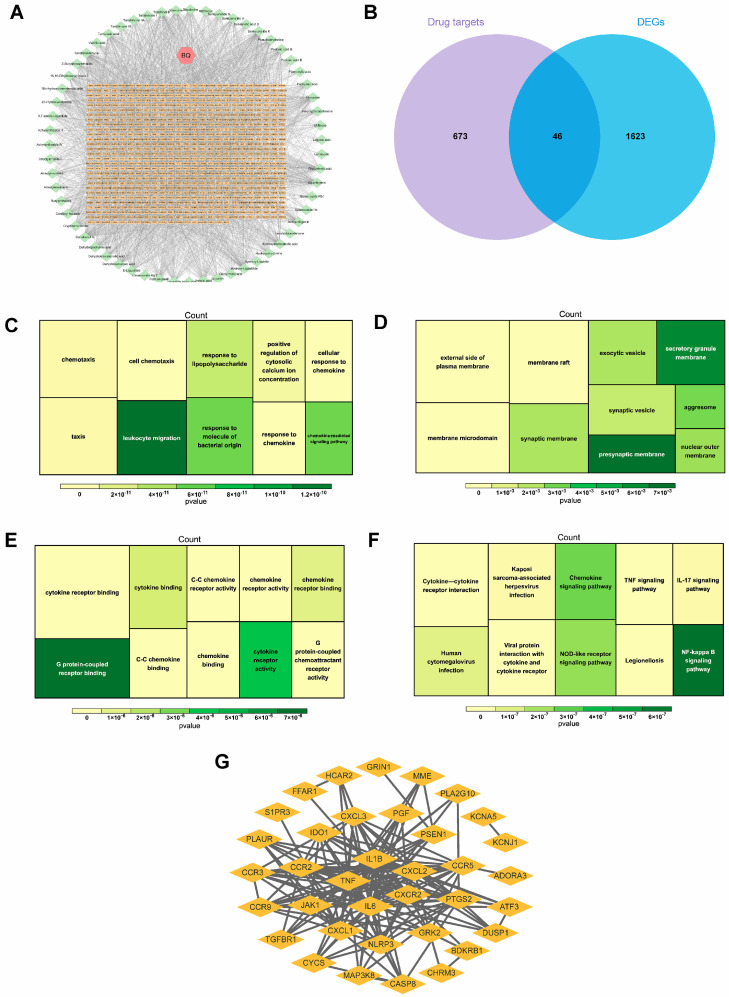
Identification and interaction of potential candidate genes in gout. (**A**) Bi-Qi capsule–active ingredient–target network. (**B**) Venn plot of the intersection of DEGs and Bi-Qi capsules. (**C**–**E**) GO functional enrichment analysis of candidate genes, including biological processes, cellular components, and molecular functions. (**F**) KEGG functional enrichment analysis of candidate genes. (**G**) Protein–protein interaction (PPI) network for interaction of candidate genes with confidence score > 0.4.

A total of 59 active chemical components and 719 related targets from Bi-Qi capsules were screened (Figure 3A). By intersecting the identified targets of Bi-Qi capsules and 1669 DEGs, 46 candidate genes were discovered (Figure 3B). GO enrichment analysis revealed that candidate genes were primarily associated with 30 related biological processes, cellular components, and molecular functions, such as leukocyte migration, cell chemotaxis, response to lipopolysaccharides and molecules of bacterial origin, G protein-coupled receptor binding, and chemokine receptor activity and binding (Figure 3C–E). KEGG enrichment analysis revealed that candidate genes take part in the NF-kappa B, chemokine, NOD-like receptor, human cytomegalovirus infection, TNF, and IL-17 signaling pathways (Figure 3F). The data from the PPI network were input into Cytoscape 3.7.2 software in a TSV file format, and an intersection target correlation network diagram was generated, as shown in Figure 3G. CXCR2, CXCL2, IL1B, TNF, and IL6 were identified as core genes with the highest number of interactions, suggesting their central roles in the PPI network.

### 2.3. Causal Relationship of Candidate Targets and Gout

**Figure 4:**
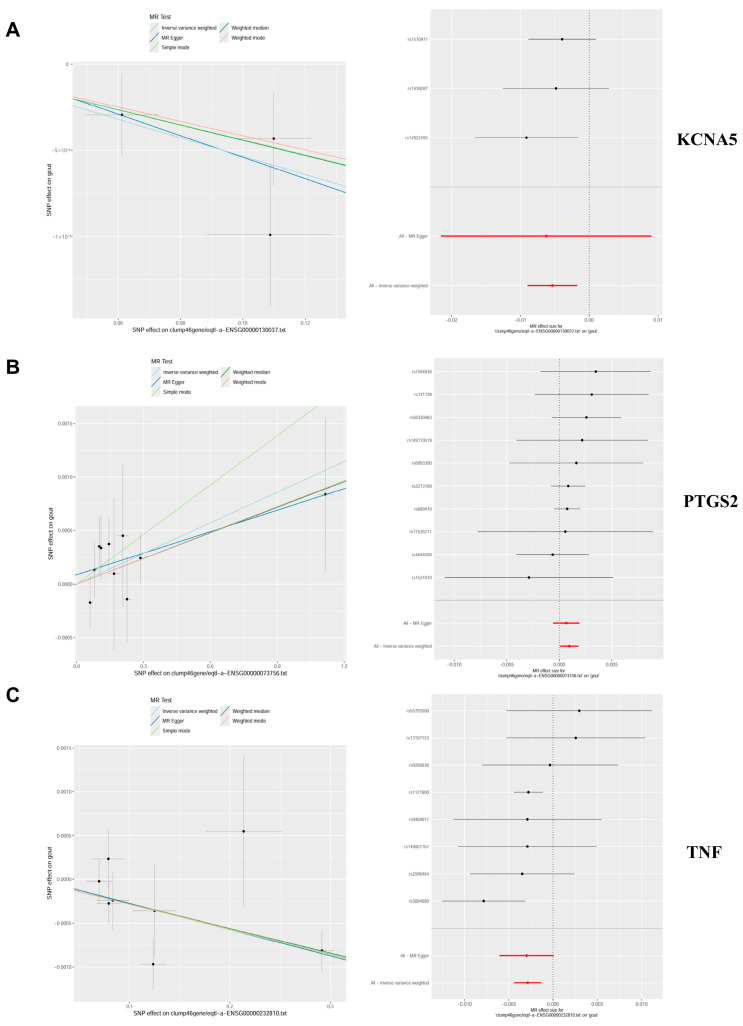
Mendelian randomization (MR) analysis and expression analysis of three crucial targets in gout. (**A**–**C**) The scatter plots and forest plots of the MR analysis for KCNA5, PTGS2, and TNF.

Genetic causality between candidate targets and gout was assessed through bidirectional MR analysis. In total, three candidate targets including KCNA5, PTGS2, and TNF were identified as having genetic causality with gout and were selected for further analysis as critical targets. Compared with the normal group, the PTGS2 and TNF expression levels were dramatically higher in the gout group, and the expression of KCNA5 was prominently lower in the gout group (Figure S1A). In IVW analysis (Table S2), the results of the forest plots and scatter plots suggested that PTGS2 (*p* < 0.05, OR = 1.0010) promotes the pathological development of gout, with elevated PTGS2 levels increasing the risk of gout development. Conversely, KCNA5 (*p* < 0.05, OR = 0.9947) and TNF (*p* < 0.05, OR = 0.9971) exhibited opposing effects (Figure 4A–C). The funnel plot presented a symmetrical state, suggesting that the MR results were stable and robust (Figure S1B). Moreover, the reliability of the MR results was analyzed, and a series of sensitivity tests was conducted. In the horizontal pleiotropy test, there was no statistical difference (*p* > 0.05) in light of the MR–Egger intercept, indicating the absence of confounding factors. In the heterogeneity test, Cochrane’s Q value was much greater than 0.05 without heterogeneity among IVs (Table S3). In the LOO analysis, it was discovered that no single SNP strongly altered the holistic forest plot of KCNA5, PTGS2, and TNF for gout (Figure S1C).

**Figure 5:**
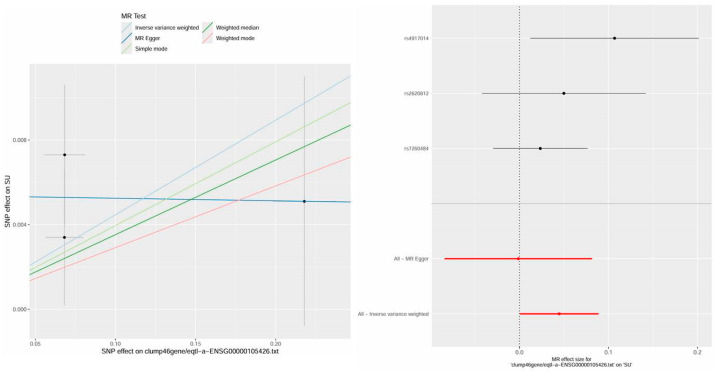
The scatter plot and forest plot of the MR analysis and expression analysis for PTPRS in hyperuricemia (HUA).

In addition, this study also found a causal relationship between PTPRS and HUA, providing more theoretical perspectives on the mechanism of Bi-Qi capsules in treating gout. The results display a significant causal relationship between PTPRS and HUA in IVW analysis (*p* < 0.05, OR = 1.0457), which increases the risk of gout with increasing levels (Table S4 and Figure 5). After sensitivity analysis, it was suggested that the MR results between PTPRS and HUA are reliable (Table S5 and Figure S2).

### 2.4. Cell Annotation, Differential Expression, and Enrichment Analysis

**Figure 6:**
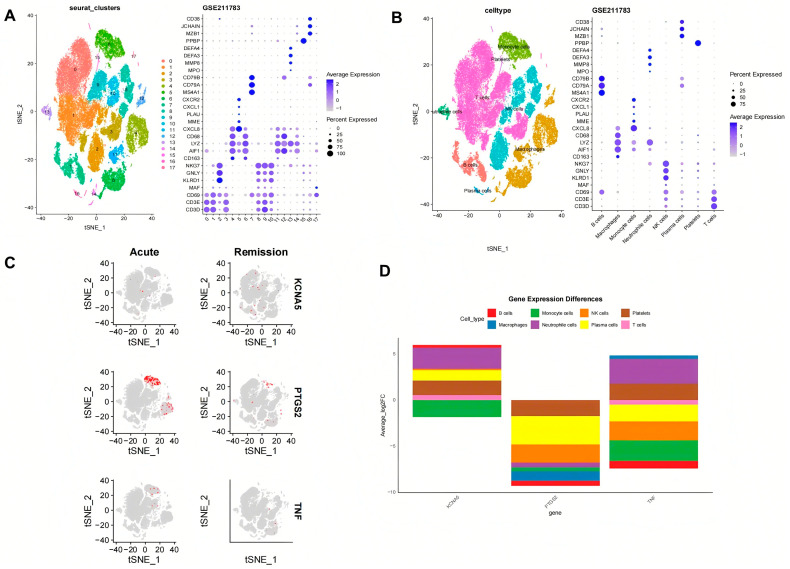
The crucial targets’ expression in different blood cell types of gout. (**A**) t-Distributed Stochastic Neighbor Embedding (TSNE) plot showing 18 clusters at a resolution of 0.2. (**B**) Dot plot showing the crucial genes of 8 different cell types with a renamed TSNE plot. (**C**,**D**) The crucial targets’ expression status in different cell types in the acute phase and remission phases.

Through scRNA-seq and gene labeling, a total of 53,455 cells (27,333 cells in acute phase and 26,122 cells in remission phase) and 21,034 genes were identified. These cells were divided into eighteen cell clusters and eight cell types under the condition of dims = 20 and resolution = 0.2. There is no obvious mixed intersection among the 18 cell clusters (Figure 6A), mainly comprising B cells, monocyte cells, NK cells, platelets, macrophages, neutrophile cells, plasma cells, and T cells (Figure 6B). Meanwhile, the expression status of marker genes across different cell clusters and cell types was also analyzed. In the acute phase, it was found that KCNA5 expression is mainly focused on monocyte cells, T cells, NK cells, and B cells, PTGS2 expression is mainly focused on monocyte cells and macrophage cells, and TNF expression is mainly focused on monocyte cells, T cells, and NK cells. In the remission phase, the expression patterns of the genes were as follows: KCNA5 expression widely exists in various types of cells, PTGS2 expression is mainly focused on monocyte cells, T cells, neutrophile cells, and macrophages, and TNF expression is predominantly localized to macrophages, T cells, and NK cells (Figure 6C). During the evolution from the acute phase to the remission phase of gout (Figure 6D), KCNA5 in B cells and neutrophil cells was raised significantly, PTGS2 in B cells, macrophages, and monocytes was decreased significantly, and TNF in B cells and monocytes was also decreased significantly.

**Figure 7:**
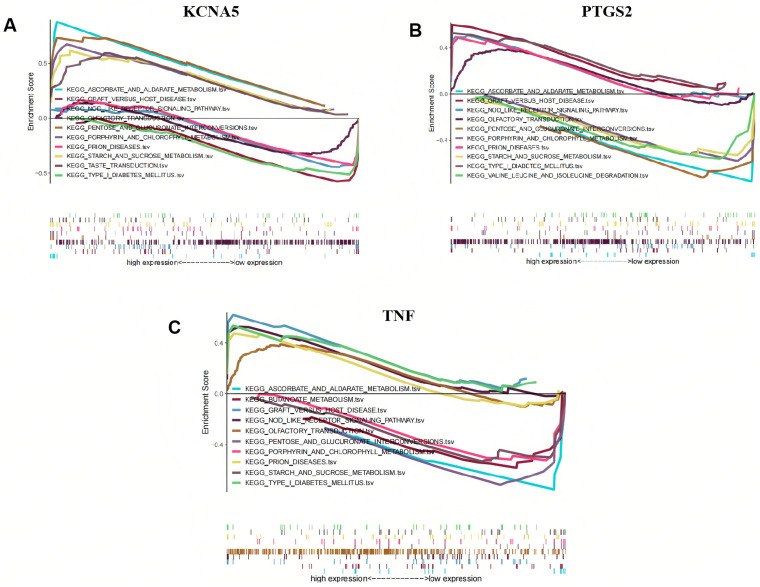
Gene set enrichment analysis (GSEA) of crucial targets. (**A**) KCNA5, (**B**) PTGS2, and (**C**) TNF.

KCNA5, PTGS2, and TNF were found to significantly participate in ascorbate and aldarate metabolism, graft-versus-host disease, the NOD-like receptor signaling pathway, olfactory transduction, pentose and glucuronate interconversions, porphyrin and chlorophyll metabolism, prion diseases, starch and sucrose metabolism, and type I diabetes mellitus (Figure 7A–C). Furthermore, KCNA5, PTGS2, and TNF are also involved in the taste transduction process, valine, leucine, and isoleucine degradation, and butyrate metabolism, respectively.

### 2.5. Regulatory Mechanism of Crucial Targets on Other Molecules

**Figure 8:**
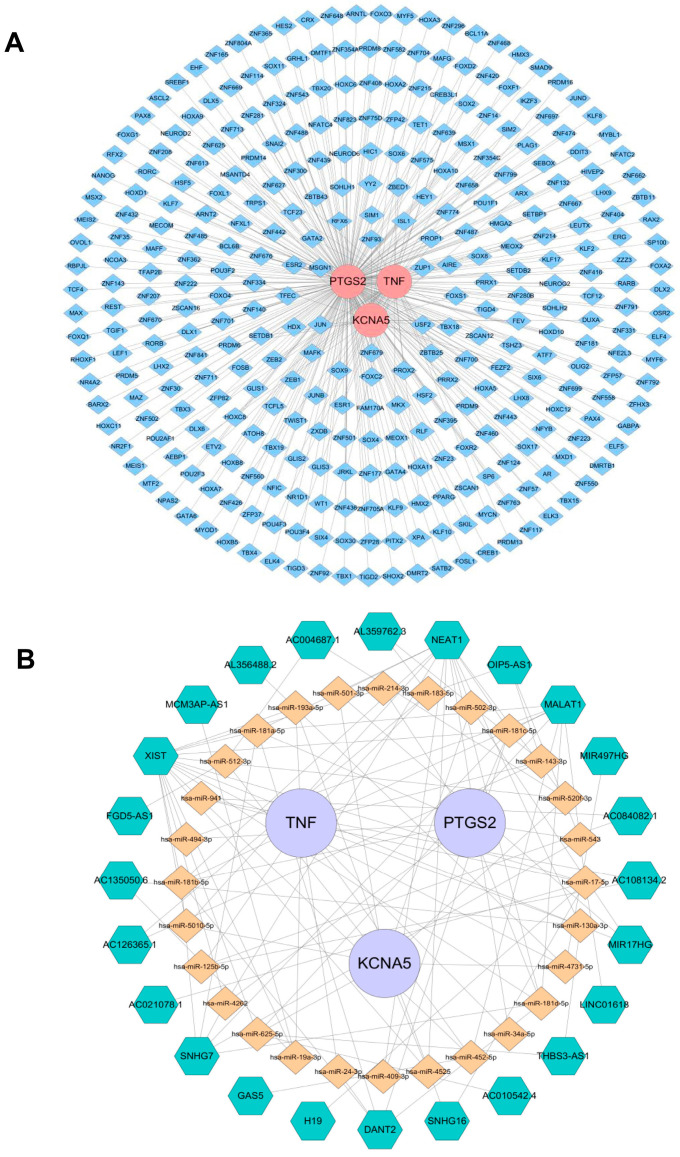
Regulatory networks of three crucial targets. (**A**) Transcription factors (TFs)–crucial targets network. Pink represents the target genes, and blue represents TFs. (**B**) The competitive endogenous RNA network targeting crucial targets. Purple represents key target genes, yellow represents miRNAs, and green represents lncRNAs.

In an effort to investigate the potential mechanism of KCNA5, PTGS2, and TNF in depth, the targeted TFs, miRNAs, and lncRNAs of crucial targets were screened and predicted by online public databases. In total, 327 TFs were identified as being associated with crucial targets (Figure 8A). Among them, eight TFs such as HDX, RFX6, SIM1, and FOXC2 collectively regulate the crucial targets KCNA5 and PTGS2, and eighteen TFs such as MSGN1, USF2, PROX2, and MAFK together regulate the crucial targets PTGS2 and TNF. JUN simultaneously regulates the three crucial targets mentioned above. Moreover, an “mRNA–miRNA–IncRNA” network that contains 28 miRNAs, 24 lncRNAs, KCNA5, PTGS2, and TNF is shown in Figure 8B, in which MALAT1, NEAT1, and XIST regulate KCNA5, PTGS2, and TNF through various miRNAs, respectively.

### 2.6. Active Ingredient–Crucial Target Binding Capacity Analysis

**Figure 9:**
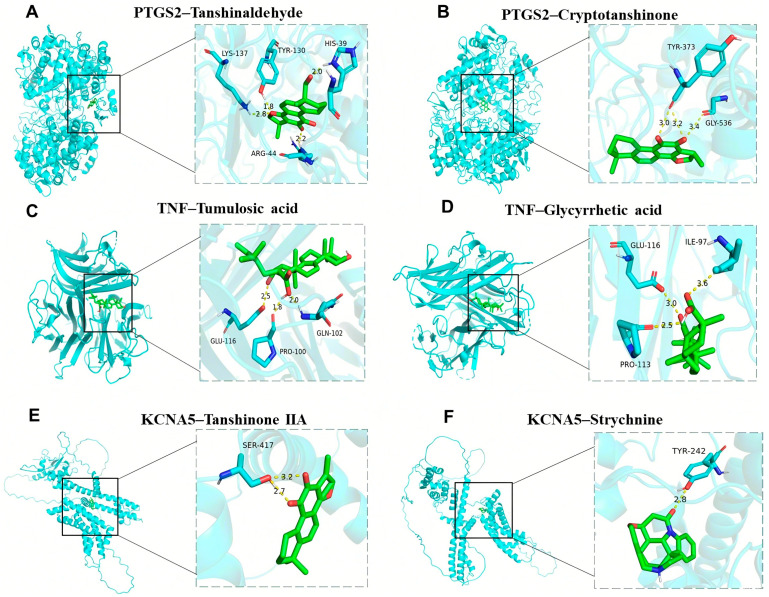
Molecular docking results of the main drug compounds and protein targets. (**A**) PTGS2 docking with Tanshinaldehyde. (**B**) PTGS2 docking with Cryptotanshinone. (**C**) TNF docking with Tumulosic acid. (**D**) TNF docking with Glycyrrhetic acid. (**E**) KCNA5 docking with Tanshinone IIA. (**F**) KCNA5 docking with Strychnine.

Molecular docking analysis verified the binding ability of crucial targets to 27 active components, such as strychnine, tanshinaldehyde, ligustilide, senkyunolide, glycyrrhetic acid, pachymic acid, ferulic acid, and tanshinone, of Bi-Qi capsules. The binding energies of these active ingredients to the targets are all below −5 kcal/mol, indicating they have strong binding affinity. Among them, the binding energy between tanshinaldehyde and PTGS2 is the lowest, reaching −10.2 kcal/mol; the binding sites are the amino acids LYS137, TYR130, HIS39, and ARG44 (Figure 9A). Next is the binding energy between cryptotanshinone and PTGS2, reaching −9.6 kcal/mol, and the binding sites are the amino acids TYR373 and GLY536 (Figure 9B). In addition, the binding energies of tumulosic acid and glycyrrhetic acid with TNF are −8.7 kcal/mol and −8.2 kcal/mol (Figure 9C,D), respectively. The binding energies of tanshinone IIA and strychnine with KCNA5 are −7.8 kcal/mol and −7.7 kcal/mol (Figure 9E,F), respectively. These results support these ingredients as potential effective ingredients in Bi-Qi capsules for treating gout.

## 3. Discussion

This study found that there are 59 active chemical ingredients and 719 related targets in Bi-Qi capsules. Combined with a literature review [24,25,26,27], we identified some important active ingredients, such as brucine, strychnine, atractylenolide, pachymic acid, tanshinone, notoginsenoside, ferulic acid, achyranthoside, and liquiritin. Forty-six candidate targets were obtained from the intersection of drug targets and disease DEGs. The results of GO enrichment analysis showed that biological processes have the most bearing on biological reactions, such as leukocyte migration, lipopolysaccharide and bacterial reactions, chemokines, and cellular reactions, which provides a theoretical basis for further elucidating the pharmacodynamic mechanism of Bi-Qi capsules in treating gout. The results of KEGG enrichment analysis highlighted multiple signaling pathways during the treatment process of gout with Bi-Qi capsules. It is worth noting that inflammatory pathways, such as the NF kappa B, chemokine, NOD-like receptor, TNF, and IL-17 signaling pathways, as well as infection-related pathways such as human cytomegalovirus infection, Kaposi sarcoma-associated herpesvirus infection, and legionellosis, play an important role during this process. To gain a deeper insight into the interactions between the candidate targets of the drug, we constructed a PPI network in which CXCR2, CXCL2, IL1B, TNF, and IL6 are considered central nodes, exhibiting a higher number of interactions.

Dual-sample MR analysis based on the GWAS data of candidate genes (exposure factors) and gout (outcome factors) was employed to perform causal relationship research. The results of the MR analysis with good sensitivity suggested a causal relationship between candidate genes (KCNA5, PTGS2, and TNF) and gout, in which high levels of PTGS2 increase the hazard of gout, while KCNA5 and TNF can diminish the occurrence of gout. The above-mentioned genes are considered as crucial targets for further in-depth research on the treatment of gout with Bi-Qi capsules.

As a COX-2 encoding protein, PTGS2 serves as a pivotal enzyme in the biosynthesis of prostaglandins (PGs) from arachidonic acid. COX-2 promotes the expression of proinflammatory cytokines and growth factors within the brain and spinal cord, participating in the neural transmission of pain and fever [28]. It was reported that monosodium urate (MSU) crystals, as the etiological agent of gout, specifically induce COX-2 overexpression in human monocytes [29]. Furthermore, COX-2 overexpression and PG production have been observed in arthritis and inflammatory bowel disease [30]. Our research showed a similar trend of increased PTGS2 expression in gout patients, suggesting that PTGS2 plays a significant role in gout-associated inflammation and pain and has the potential to be regarded as a diagnostic biomarker for gout [31]. Kv channels are involved in the control of cell excitability, contributing to the regulation of electron migration, in which KCNA5 encodes Kv1.5 with various cell functions, such as cell migration, proliferation, and apoptosis [32,33]. KCNA5 expression occurs in response to oxidative stress mediated by the Sp1 transcription factor [34]. Certain research has demonstrated that a delayed rectifier K+ channel has the ability to re-establish the resting membrane potential in beta cells following depolarization, thus playing a role in regulating insulin secretion [35]. GIP and GLP-1 decreased the rate of cell death in INS-1 β cells (clone 832/13) that overexpressed Kv1.5. Additionally, when endogenous Kv1.5 was knocked down through RNAi-mediated interference, the apoptotic death of β cells was reduced. Both GIP and GLP-1 promoted the phosphorylation and acetylation of Kv1.5 and its Kvβ2 protein subunit, which strengthened their interaction [36]. Currently, although there is currently a lack of research reports on the relationship between KCNA5 and gout, considering that the emergence of gout is related to glucose metabolism levels, combined with the results of this study, it can be speculated that KCNA5 has the potential to inhibit gout attacks. TNF, as one of the key mediators of the immune system, belongs to the tumor necrosis factor superfamily, which exerts biological effects depending on two main receptors, TNFR1 and TNFR2. TNFRs are widely expressed in different types of cells, such as immune cells, endothelial cells, and fibroblasts [37]. The binding of TNF to receptors can activate various signaling pathways involved in the physiological and pathological processes of cells, including the NF-κB pathway, JNK pathway, p38 MAPK pathway, and others [38,39]. TNF-α is a pivotal component of the normal immune response, which can activate the immune system for regulation [40]. Research has found that under normal circumstances, low levels of TNF-α in plasma have physiological functions such as killing or inhibiting tumor cells, enhancing the phagocytic ability of neutrophils, resisting infections, and promoting cell proliferation and differentiation [41,42]. However, the abnormal secretion of TNF-α is harmful and may lead to rheumatoid arthritis, inflammatory bowel disease, psoriatic arthritis, ankylosing spondylitis, psoriasis, and noncommunicable uveitis [43,44]. PTPRS is a protein coding gene related to phosphatase activity and transmembrane receptor protein tyrosine phosphatase activity. Diseases associated with PTPRS include NK-cell enteropathy and papillary tumors of the pineal region; the related pathways are signaling by NTRKs and protein–protein interactions at synapses. Some research has found that PTPRS has been identified as the most up-regulated gene in gout patients [45]. The findings reported in the literature align with the results of our study.

scRNA-seq data were obtained from the GEO database to verify the distribution of crucial targets. The results showed that the three key targets involved eighteen cell clusters, mainly distributed across eight types of cells, including B cells, monocyte cells, NK cells, platelets, macrophages, neutrophile cells, plasma cells, and T cells. The systemic immune and inflammatory responses of the body gradually decrease during the evolution of gout from the acute phase to the remission phase, though the KCNA5 level in B cells and neutrophil cells was raised, and PTGS2 and TNF in B cells, macrophages, and monocytes were decreased. Through GSEA, we can identify signaling pathways or functional modules that are active under specific biological conditions, providing important clues for disease mechanisms, drug target discovery, and biomarker development. This method not only improves the understanding of genomic data but also promotes the development of precision medicine and personalized treatment.

In our study, crucial targets in the treatment of gout are mainly related to carbohydrate metabolism, nerve conduction, and the inner immune pathway. For every 60 μ mol/L increase in the serum uric acid (SUA) level, the danger of type 2 diabetes increases by 17%. Patients with hyperuricemia exhibit a 95% increased risk of diabetes compared to those with normal blood uric acid levels [46]. HUA damages pancreatic beta cells, leading to increased blood sugar levels through a reduction in insulin secretion and an increased burden on pancreatic beta cells. At the same time, it also affects the metabolism of blood sugar in the kidneys and induces various diseases such as gouty arthritis and urinary tract infections [47]. Ascorbic acid and aldosterone metabolism are important carbohydrate metabolic pathways that protect cells from oxidative damage. There are various biological functions which could suppress protein glycosylation though the competitive inhibition of non-enzyme-mediated glycosylation and hinder the production of oxygen free radicals and lipid peroxidation products to reduce the damage caused by renal hyperoxidative stress [48,49,50,51]. Compared with the control group, the disruption of the metabolic pathways of ascorbic acid and alginate in both the hyperuricemia and gout groups is consistent with our research findings [52]. Taste conduction plays an important role in metabolic diseases by binding olfactory receptors located on tissues with odor molecules, bioactive factors, and other substances. For example, the activation of olfactory receptor 1A2 by citral results in a reduction in the phagocytic ability of alveolar macrophages and the promotion of the release of pro-inflammatory cytokines. Olfactory receptor 2 activated by octanal positively regulates the inflammatory response and aggravates atherosclerosis [53,54,55]. Some studies have reported that the shared loci are concentrated in the olfactory receptor pathway. HNF4A regulates the expression of multiple genes, among which is hepatocyte nuclear factor 1 alpha, a transcription factor that modulates the expression of various hepatic genes. This particular gene may be involved in the development of the liver, kidneys, and intestines. Genetic heterogeneity at the HNF4A locus results in different genetic variants that confer risk for both hyperuricemia and chronic kidney disease, hinting at the involvement of distinct pathways. These findings offer insights into the shared underlying mechanisms of hyperuricemia/gout and chronic kidney disease [56]. The NOD-like receptor signaling pathway plays a crucial role in pathogen recognition and the innate immune response by specifically recognizing receptor family patterns. As a typical representative of the NOD-like receptor protein family, the accumulation of MSU crystals in the joints of gout patients can trigger the formation of NLRP3 inflammasomes. NLRP3 inflammasomes activate IL-1β by activating caspase-1 and then activate the NOD receptor family signaling pathway to induce gout inflammation. The abnormal expression and activity of NLRP3 inflammasome components are frequently observed in patients with gouty arthritis and animal models of the disease [57,58].

We predicted 327 TFs, 28 miRNAs, and 24 lncRNAs targeting KCNA5, PTGS2, and TNF; then, we constructed ceRNA networks which helped us to further investigate gene regulatory mechanisms and reveal the occurrence and development patterns of gout, which offers innovative concepts and approaches for gout management. In molecular docking and dynamics simulations, active ingredients of KCNA5, PTGS2, and TNF were found, including tanshinone IIA, strychnine, tanshinaldehyde, cryptotanshinone, tumulosic acid, and glycyrrhetic acid, which possess good binding properties. Therefore, we believe that KCNA5, PTGS2, and TNF may perform a key function in the treatment of gout with Bi-Qi capsules. Eight TFs, such as HDX, RFX6, SIM1, and FOXC2, together regulate the crucial targets KCNA5 and PTGS2; eighteen TFs, such as MSGN1, USF2, PROX2, and MAFK, together regulate the crucial targets PTGS2 and TNF. JUN simultaneously regulates the three crucial targets mentioned above. MALAT1, NEAT1, and XIST regulate KCNA5, PTGS2, and TNF through various miRNAs, respectively.

Compared to conventional network pharmacology, the method in this research can better reflect the characteristics of the disease. By utilizing multidimensional bioinformatics strategies such as network pharmacology, MR, and scRNA-seq analysis, the crucial targets of Bi-Qi capsules for gout are screened and validated, which makes the evidence stronger. Not only does this promote the refined development of traditional Chinese medicine and drug development but it also helps to clarify the relevant treatment mechanisms and identify potential clinical biomarkers.

In the future, we can use the results of this study to develop new drugs from Bi-Qi capsules, including reducing the quantity of plant materials, novel combinations of active ingredients, and key target modulators with the aim of improving patient compliance and achieving better treatment outcomes. Furthermore, new clinical applications of Bi-Qi capsules can also be discovered. The common single-cell analysis emphasizes the comparison of differences between normal individuals and patients. This study focuses on the differential analysis of the acute attack and remission stages of gout. From a clinical perspective, patients often seek medical assistance during acute attacks of gout to prevent further development. Understanding the development and evolution of diseases is more conducive to rational clinical medication and has greater clinical relevance and practical value.

However, several limitations need to be considered. It is the intrinsic trait of network pharmacology research that some active ingredients and drug targets might not have been encompassed within public databases, which makes it difficult to clarify whether there are synergistic advantages or adverse effects between substances, and some isolated important active ingredients may have been ignored in this study. Although MR design was used to reduce the influence of confounding factors, there may still be other potential factors that could affect the results. Due to differences in genetic background and linkage disequilibrium patterns, changes in demographic background may introduce potential biases when calculating the effects of MR. It is essential to generalize the outcomes to individuals of diverse racial backgrounds to guarantee the broader applicability of the research discoveries. Furthermore, utilizing blood samples solely from gout patients may introduce biases and constraints in sample selection, which limits the ability of this research to conduct a comprehensive analysis of various subtypes and severity levels of gout. Despite multidimensional analysis and screening, the potential drug targets identified in this study do not guarantee reliable effectiveness in real-world clinical settings. Further experimental validation and clinical trials are required to ascertain the actual therapeutic promise of these key targets.

## 4. Materials and Methods

### 4.1. Study Design

In this research, a multidimensional data analysis strategy integrating conventional bioinformatics methods with MR studies of drug targets was employed to elucidate the therapeutic potential of Bi-Qi capsules in the treatment of gout. Based on data from the Gene Expression Omnibus (GEO) database and other online public databases, the potential mechanism of Bi-Qi capsules in treating gout was primarily identified by network pharmacology. The MR study of drug targets was subsequently utilized to explain the causal relationship between the crucial targets of Bi-Qi capsules and gout. Then, we further conducted single-cell RNA sequencing and gene set enrichment analysis and constructed regulatory networks of transcription factors (TFs), miRNAs, and lncRNAs for crucial targets. Finally, molecular docking technology was applied to evaluate the binding ability of the active chemicals of Bi-Qi capsules to crucial targets. The data sources for this study are listed in Table S1.

### 4.2. Bi-Qi Capsule Characterization

Based on the instructions of Bi-Qi capsules, the dosage is to take 4 pills each time, 2–3 times a day. The levels of two key active ingredients, brucine and strychnine, were tested, both of which are essential for the medication’s efficacy. According to the preliminary research of our team, the average levels of brucine and strychnine are 0.16 mg/grain and 0.28 mg/grain, respectively. HPLC and MS fingerprints of Bi-Qi capsules are shown in Figure S3. The composition of some chromatographic peaks has been confirmed, such as strychnine, brucine, liquiritin, ferulic acid, rosmarinic acid, ginsenoside Rg1, and salvianolic acid B [59,60].

### 4.3. Bi-Qi Capsule Active Ingredients and Related Gene Collection

Bi-Qi capsule components were collected from China national knowledge infrastructure (CNKI) references and online technology platforms such as the TM-MC2.0 database (https://tm-mc.kr/ (accessed on 3 September 2024)) and traditional Chinese medicine system pharmacology (TCMSP, https://www.tcmsp-e.com/tcmsp.php (accessed on 8 September 2024)). Compared to all TCM databases, TM-MC2.0 provides the largest set of information on medicinal compounds listed in Korean, Chinese, and Japanese pharmacopoeias. Specifically, TM-MC2.0 manually extracts and organizes information on the compounds in medicinal herbs from the PubMed literature, adding marker compounds in medicinal herbs and many newly discovered compounds. Relevant targets were matched to available components, mainly identified with Swiss Target Prediction (http://swisstargetprediction.ch/ (accessed on 14 September 2024)) and STITCH (http://stitch.embl.de/ (accessed on 19 September 2024)).

### 4.4. Differentially Expressed Gene Collection

DEGs in the gout groups and normal groups were identified from the microarray data of GSE160170 in the GEO database (http://www.ncbi.nlm.nih.gov/geo/ (accessed on 6 October 2024)) and were then screened under the criteria of|log_2_FC| > 1 and *p* value < 0.05. Candidate targets were determined by the intersection of DEGs and Bi-Qi capsule-related genes.

### 4.5. Functional Enrichment and Gene Interaction Analysis

To better interpret the potential biological function of DEGs and candidate targets, GO and KEGG enrichment analyses were used. Then, a PPI network with a confidence score of >0.4 was established by searching the STRING database (https://string-db.org/ (accessed on 10 October 2024)) to determine the correlation among candidate targets.

### 4.6. MR Analysis

Multiple MR methods were employed to explore and demonstrate the causal relationship between candidate targets and gout, such as Inverse Variance Weighted (IVW), Mendelian randomization–Egger (MR–Egger), Simple Mode, Weighted Median (WM), and Mendelian Randomization Pleiotropy RESidual Sum and Outlier (MR-PRESSO), with the IVW method dominating [19,61,62]. Candidate targets were deemed as exposure factors; single nucleotide polymorphisms (SNPs) dramatically integrated with exposure factors were selected as instrumental variables (IVs) under the condition of a *p* value of less than 1 × 10^−5^ and excluding linkage disequilibrium and weak IVs, and then gout (GSE211783) was used as an outcome factor for MR analysis. A *p* value < 0.05 in the IVW method indicates a potential causal relationship. In addition, odds ratios (ORs) were calculated; exposure factors promote the outcome factor with an OR value ≥ 1, and exposure factors inhibit the outcome factor with an OR value < 1. A sensitivity analysis was conducted to evaluate the reliability of the MR results through horizontal pleiotropy, heterogeneity, and Leave-One-Out (LOO) analysis [63]. Cochran’s Q test was applied to assess the heterogeneity of the SNPs. A *p* value > 0.05 indicates a lack of substantial heterogeneity. The MR–Egger regression equation was used to evaluate the horizontal pleiotropy of genetic tools, and *p* > 0.05 indicates the absence of horizontal pleiotropy. MR-PRESSO outlier detection was employed to check horizontal pleiotropy and achieve LOO analysis by removing or reducing the weight of outliers. In order to determine that no SNPs have a real influence on the analysis results, we adopted a method of sequentially deleting each SNP and comparing the results of the IVW method with those of each deleted SNP, where an FDR < 0.05 was considered significant. Scatter plots, forest plots, and funnel plots were used to exhibit the results.

Moreover, this research also used the MR method to confirm the causal relationship between candidate genes and HUA, with candidate targets as exposure factors and HUA (GCST008972) as the outcome factor. The analysis method and the operational steps are the same as described above.

### 4.7. Single-Cell RNA Sequencing Analysis

To elucidate the differential expression patterns of critical targets across various cell types, single-cell RNA sequencing (scRNA-seq) was employed to identify cell types and examine the expression of these targets. The scRNA-seq data of crucial targets were downloaded from GEO (Registration number: GSE211783), and then the Seurat package was applied for further analysis. The quality control process ensured the following conditions and operations: (1) selecting genes that need to be expressed in at least three cells; (2) ensuring each cell expresses between 200 and 8000 genes; and (3) limiting the proportion of mitochondrial genes to less than 25%. The RNA sequencing data were normalized to TPM and subsequently scaled using the NormalizeData and ScaleData functions, and dimensionality reduction was then performed by PCA to facilitate the analysis of high-dimensional data. After these samples were eliminated from batch processing effects and merged together using the Harmony package, all cells were clustered using the Find Clusters function (resolution = 0.2) and visualized by the t-Distributed Stochastic Neighbor Embedding (TSNE) method. By searching for the biomarkers of different cells, the corresponding cell types were labeled. To investigate whether crucial genes are expressed differently in specific blood cell types of patients with gout, differential expression analysis stemming from the Wilcoxon Ran-Sum Test was employed to compare the protein expression levels among different cell types. Genes with an average log 2-fold change greater than 1 and *p* < 0.05 were regarded as differential expressed genes in a certain cell type.

### 4.8. Gene Set Enrichment Analysis

Compared with traditional enrichment analyses such as GO and KEGG based on hypergeometric or Fisher’s Exact Test, GSEA can avoid the shortcomings of traditional methods. GSEA addresses the challenges associated with genes exhibiting small expression changes and provides a more comprehensive understanding of the overall trend of gene sets, rather than focusing solely on differentially expressed genes. In this study, GSEA version 4.3.3 was employed in conjunction with the GSE160170 dataset to conduct enrichment analysis on the expression of critical genes; the number of permutations was set to 1000, and other parameters were set as default. Subsequently, the upward or downward trend of the entire pathway was visually displayed using the R software ggplot2 package.

### 4.9. Construction of Interaction Network of Crucial Targets and Other Molecules

Using the ChEA3 database (https://maayanlab.cloud/chea3/ (accessed on 21 October 2024)), we predicted transcription factors (TFs) associated with crucial targets under the condition of a score > 500. Then, miRNAs of crucial targets were mainly obtained under the screening criteria clipExpNum > 6 through the Starbase database (https://starbase.sysu.edu.cn/index.php (accessed on 22 October 2024)). Furthermore, IncRNAs related to miRNAs were predicted using this database with a screening criterion of clipExpNum > 10. Cytoscape software (version 3.8.2) was utilized to construct the “TF–crucial targets” network and “lncRNA–miRNA–mRNA” network.

### 4.10. Molecular Docking

For the purpose of predicting the binding ability between the active ingredients of Bi-Qi capsules and crucial targets, molecular docking analysis was conducted. Firstly, the 2D structures of active ingredients were acquired from the PubChem database (https://pubchem.ncbi.nlm.nih.gov/ (accessed on 24 October 2024)), and then they were converted into 3D structures by Chem3D software (version 22.0.0). After downloading the PDB format of crucial targets (https://www.resb.org/ (27 October 2024)), water and ligands were removed from the crucial target protein via PyMOL software (version 2.4.0). In addition, if the database did not include the protein structure of crucial targets, the alphafold database (https://www.alphafold.com/ (accessed on 27 October 2024)) was used to search for the protein structure. Using Autodock Tools 1.5.6 for optimization, such as hydrogenation and charging, crucial target proteins and active ingredients were converted into “pdbqt” files for molecular docking with Autodock Vina 1.1.2. The binding conformation with the lowest free binding energy value was chosen as the optimal conformation. The molecular docking result was visualized with PyMOL and Discovery Studio software (version 2.1).

### 4.11. Statistical Analysis

Statistical analysis was performed using R software version 4.1.3, with the utilization of the “Mendelian Randomization” and “MR-PRESSO” packages for the MR analysis. Statistical significance was set at a threshold of *p* < 0.05 to indicate potential causal effects.

## 5. Conclusions

In this research, we explore the therapeutic potential of Bi-Qi capsules in the treatment of gout by discovering crucial drug targets. It is indicated that Bi-Qi capsules could modulate various biological processes in the treatment of gout, such as the inflammatory response, immune defense, and metabolic regulation. KCNA5, PTGS2, and TNF were identified as crucial targets for Bi-Qi capsules in the treatment of gout, which might provide new evidence for the future clinical application of Bi-Qi capsules.

## Data Availability

The original data presented in this study are mainly openly available in the GWAS database (https://www.ebi.ac.uk/gwas/ (accessed on 14 October 2024)).

## Supplementary Materials

The following supporting information can be downloaded at https://www.mdpi.com/article/10.3390/ph18050618/s1. Figure S1: (A) The expression levels of KCNA5, PTGS2, and TNF in the normal and gout groups. (B) Funnel plots of the MR analysis for three crucial targets in gout. (C) Leave-One-Out (LOO) analysis of the MR analysis for sensitivity analyses of three crucial targets in gout. Figure S2: (A) Funnel plots of the MR analysis for PTPRS and HUA. (B) LOO analysis of the MR analysis for sensitivity analyses of PTPRS and HUA. Figure S3: HPLC(A) and MS (B) fingerprints of Bi-Qi Capsules. Table S1: Data sources for this research. Table S2: MR analysis of causal relationships of crucial targets and gout. Table S3: Results of heterogeneity test and horizontal pleiotropic test of crucial targets and gout. Table S4: MR analysis of causal relationship of PTPRS and hyperuricemia (HUA). Table S5: Results of heterogeneity test and horizontal pleiotropic test of PTPRS and HUA.

## Abbreviations

The following abbreviations are used in this manuscript:

MR	Mendelian randomization
GSEA	Gene set enrichment analysis
SUA	Serum uric acid
GEO	Gene Expression Omnibus
SNPs	Single nucleotide polymorphisms
LOO	Leave One Out

## References

[1] Dalbeth N., Te Karu L., Stamp L.K. (2024). Gout and its management. Intern. Med. J..

[2] Scuiller A., Pascart T., Bernard A., Oehler E. (2020). La maladie goutteuse [Gout]. Rev. Med. Interne.

[3] Dehlin M., Jacobsson L., Roddy E. (2020). Global epidemiology of gout: Prevalence, incidence, treatment patterns and risk factors. Nat. Rev. Rheumatol..

[4] Danve A., Neogi T. (2020). Rising Global Burden of Gout: Time to Act. Arthritis Rheumatol..

[5] Kyu H.H., Abate D., Abate K.H., Abay S.M., Abbafati C., Abbasi N., Breitborde N.J. (2018). Global, regional, and national disability-adjusted life-years (DALYs) for 359 diseases and injuries and healthy life expectancy (HALE) for 195 countries and territories, 1990–2017: A systematic analysis for the Global Burden of Disease Study 2017. Lancet.

[6] Punzi L., Galozzi P., Luisetto R., Scanu A., Ramonda R., Oliviero F. (2024). Gout: One year in review 2023. Clin. Exp. Rheumatol..

[7] Cross M., Ong K.L., Culbreth G.T., Steinmetz J.D., Cousin E., Lenox H., Woolf A.D. (2024). Global, regional, and national burden of gout, 1990–2020, and projections to 2050: A systematic analysis of the Global Burden of Disease Study 2021. Lancet Rheumatol..

[8] Elfishawi M.M., Zleik N., Kvrgic Z., Michet C.J., Crowson C.S., Matteson E.L., Bongartz T. (2018). The Rising Incidence of Gout and the Increasing Burden of Comorbidities: A Population-based Study over 20 Years. J. Rheumatol..

[9] Cipolletta E., Tata L.J., Nakafero G., Avery A.J., Mamas M.A., Abhishek A. (2022). Association Between Gout Flare and Subsequent Cardiovascular Events Among Patients with Gout. JAMA.

[10] Disveld I.-M., Zoakman S., Jansen T.-A., Rongen G.A., Kienhorst L.-E., Janssens H.-M., Fransen J., Janssen M. (2019). Crystal-proven gout patients have an increased mortality due to cardiovascular diseases, cancer, and infectious diseases especially when having tophi and/or high serum uric acid levels: A prospective cohort study. Clin. Rheumatol..

[11] Vargas-Santos A.B., Neogi T., da Rocha Castelar-Pinheiro G., Kapetanovic M.C., Turkiewicz A. (2019). Cause-Specific Mortality in Gout: Novel Findings of Elevated Risk of Non-Cardiovascular-Related Deaths. Arthritis Rheumatol..

[12] Afinogenova Y., Danve A., Neogi T. (2022). Update on gout management: What is old and what is new. Curr. Opin. Rheumatol..

[13] Wang Q.S., Cui Y.L., Wang Y.F., Chi W. (2011). Effects of compounds from bi-qi capsule on the expression of inflammatory mediators in lipopolysaccharide-stimulated RAW 264.7 macrophages. J. Ethnopharmacol..

[14] Chen X.M., Wu J.Q., Huang Q.C., Zhang J.Y., Pen J.H., Huang Z.S., Chu Y.L., He X.H., Wang M.J., Huang R.Y. (2018). Systematic review and meta-analysis of the efficacy and safety of Biqi capsule in rheumatoid arthritis patients. Exp. Ther. Med..

[15] Zhou Y., Wang W., Tian K., Huang H., Jia M. (2021). Efficacy and safety of Biqi capsule in the treatment of knee osteoarthritis: A protocol of a randomized controlled trial. Medicine.

[16] Liu Y.X., Liu W.W., Zhao Y., Wang L.P. (2011). Clinical and pharmacological research progress of Biqi capsule in nearly three years. Chin. J. Tradit. Chin. Med..

[17] Li G., Du S., Yan S., Wang Y., Bu R., Cheng M., Zhang Y., Chen Q., Wu Y., Zhang X. (2025). Mechanism of Biqi capsules in the treatment of gout based on network pharmacology and experimental verification. J. Ethnopharmacol..

[18] Sanderson E., Glymour M.M., Holmes M.V., Kang H., Morrison J., Munafò M.R., Palmer T., Schooling C.M., Wallace C., Zhao Q. (2022). Mendelian randomization. Nat. Rev. Methods Primers.

[19] Richmond R.C., Davey Smith G. (2022). Mendelian Randomization: Concepts and Scope. Cold Spring Harb. Perspect. Med..

[20] Zhou C., Wei J., Yu P., Yang J., Liu T., Jia R., Wang S., Sun P., Yang L., Xiao H. (2023). Convergent application of traditional Chinese medicine and gut microbiota in ameliorate of cirrhosis: A data mining and Mendelian randomization study. Front. Cell. Infect. Microbiol..

[21] Lin J., Zhou J., Xu Y. (2023). Potential drug targets for multiple sclerosis identified through Mendelian randomization analysis. Brain.

[22] Chauquet S., Zhu Z., O’Donovan M.C., Walters J.-R., Wray N.R., Shah S. (2021). Association of Antihypertensive Drug Target Genes With Psychiatric Disorders: A Mendelian Randomization Study. JAMA Psychiatry.

[23] Cao Y., Yang Y., Hu Q., Wei G. (2023). Identification of potential drug targets for rheumatoid arthritis from genetic insights: A Mendelian randomization study. J. Transl. Med..

[24] Zou Y.T., Long F., Wu C.Y., Zhou J., Zhang W., Xu J.D., Zhang Y.Q., Li S.L. (2019). A dereplication strategy for identifying triterpene acid analogues in Poria cocos by comparing predicted and acquired UPLC-ESI-QTOF-MS/MS data. Phytochem. Anal..

[25] Wan M.Q., Liu X.Y., Gao H., Wang T.X., Yang Y.F., Jia L.Y., Yang X.W., Zhang Y.B. (2020). Systematic analysis of the metabolites of Angelicae Pubescentis Radix by UPLC-Q-TOF-MS combined with metabonomics approaches after oral administration to rats. J. Pharm. Biomed. Anal..

[26] Li Y.J., Wei H.L., Qi L.W., Chen J., Ren M.T., Li P. (2010). Characterization and identification of saponins in Achyranthes bidentata by rapid-resolution liquid chromatography with electrospray ionization quadrupole time-of-flight tandem mass spectrometry. Rapid Commun. Mass Spectrom..

[27] Wang C., Cai Z., Shi J., Chen S., Tan M., Chen J., Chen L., Zou L., Chen C., Liu Z. (2019). Comparative Metabolite Profiling of Wild and Cultivated Licorice Based on Ultra-Fast Liquid Chromatography Coupled with Triple Quadrupole-Time of Flight Tandem Mass Spectrometry. Chem. Pharm. Bull..

[28] Vane J.R., Bakhle Y.S., Botting R.M. (1998). Cyclooxygenases 1 and 2. Annu. Rev. Pharmacol. Toxicol..

[29] Pouliot M., James M.J., McColl S.R., Naccache P.H., Cleland L.G. (1998). Monosodium urate microcrystals induce cyclooxygenase-2 in human monocytes. Blood.

[30] Alexanian A., Sorokin A. (2017). Cyclooxygenase 2: Protein-protein interactions and posttranslational modifications. Physiol. Genom..

[31] Peng J., Gu Y., Liu J., Yi H., Ruan D., Huang H., Shu Y., Zong Z., Wu R., Li H. (2024). Identification of SOCS3 and PTGS2 as new biomarkers for the diagnosis of gout by cross-species comprehensive analysis. Heliyon.

[32] Brevnova E.E., Platoshyn O., Zhang S., Yuan J.X. (2004). Overexpression of human KCNA5 increases IK V and enhances apoptosis. American journal of physiology. Cell Physiol..

[33] Nattel S., Bourne G., Talajic M. (1997). Insights into mechanisms of antiarrhythmic drug action from experimental models of atrial fibrillation. J. Cardiovasc. Electrophysiol..

[34] Fountain S.J., Cheong A., Li J., Dondas N.Y., Zeng F., Wood I.C., Beech D.J. (2007). K(v)1.5 potassium channel gene regulation by Sp1 transcription factor and oxidative stress. American journal of physiology. Heart Circ. Physiol..

[35] Philipson L.H., Hice R.E., Schaefer K., LaMendola J., Bell G.I., Nelson D.J., Steiner D.F. (1991). Sequence and functional expression in Xenopus oocytes of a human insulinoma and islet potassium channel. Proc. Natl. Acad. Sci. USA.

[36] Kim S.J., Ao Z., Warnock G., McIntosh C.H. (2013). Incretin-stimulated interaction between β-cell Kv1.5 and Kvβ2 channel proteins involves acetylation/deacetylation by CBP/SirT1. Biochem. J..

[37] Idriss H.T., Naismith J.H. (2000). TNF alpha and the TNF receptor superfamily: Structure-function relationship(s). Microsc. Res. Tech..

[38] Zheng X., Liu H., Ma M., Ji J., Zhu F., Sun L. (2021). Anti-thrombotic activity of phenolic acids obtained from Salvia miltiorrhiza f. alba in TNF-α-stimulated endothelial cells via the NF-κB/JNK/p38 MAPK signaling pathway. Arch. Pharm. Res..

[39] Zheng L.W., Wang W.C., Mao X.Z., Luo Y.H., Tong Z.Y., Li D. (2020). TNF-α regulates the early development of avascular necrosis of the femoral head by mediating osteoblast autophagy and apoptosis via the p38 MAPK/NF-κB signaling pathway. Cell Biol. Int..

[40] Horiuchi T., Mitoma H., Harashima S., Tsukamoto H., Shimoda T. (2010). Transmembrane TNF-alpha: Structure, function and interaction with anti-TNF agents. Rheumatology.

[41] Wang Q., Huang Q., Ying X., Zhou Y., Duan S. (2024). Exploring the regulatory role of tsRNAs in the TNF signaling pathway: Implications for cancer and non-cancer diseases. Prog. Biophys. Mol. Biol..

[42] Holbrook J., Lara-Reyna S., Jarosz-Griffiths H., McDermott M. (2019). Tumour necrosis factor signalling in health and disease. F1000Research.

[43] Varfolomeev E., Vucic D. (2018). Intracellular regulation of TNF activity in health and disease. Cytokine.

[44] van Loo G., Bertrand M.-M. (2023). Death by TNF: A road to inflammation Death by TNF: A road to inflammation. Nat. Rev. Immunol..

[45] Qiu K., Zeng T., Liao Y., Min J., Zhang N., Peng M., Kong W., Chen L.L. (2022). Identification of Inflammation-Related Biomarker Pro-ADM for Male Patients with Gout by Comprehensive Analysis. Front. Immunol..

[46] Viazzi F., Leoncini G., Vercelli M., Deferrari G., Pontremoli R. (2011). Serum uric acidlevels predict new-onset type 2 diabetes in hospitalized patients with primary hypertension: The MAGIC study. Diabetes Care.

[47] Xiong Q., Liu J., Xu Y. (2019). Effects of Uric Acid on Diabetes Mellitus and Its Chronic Complications. Int. J. Endocrinol..

[48] Peng L., Chen L., Wan J., Liu W., Lou S., Shen Z. (2023). Single-cell transcriptomic landscape of immunometabolism reveals intervention candidates of ascorbate and aldarate metabolism, fatty-acid degradation and PUFA metabolism of T-cell subsets in healthy controls, psoriasis and psoriatic arthritis. Front. Immunol..

[49] Ng L.L., Ngkeekwong F.C., Quinn P.A., Davies J.E. (1998). Uptake mechanisms for ascorbate and dehydroascorbate in lymphoblasts from diabetic nephropathy and hypertensive patients. Diabetologia.

[50] Han C., Shen Z., Cui T., Ai S.S., Gao R.R., Liu Y., Sui G.Y., Hu H.Z., Li W. (2023). Yi-Shen-Hua-Shi granule ameliorates diabetic kidney disease by the “gut-kidney axis”. J. Ethnopharmacol..

[51] Dhar-Mascareño M., Cárcamo J.M., Golde D.W. (2005). Hypoxia-reoxygenation-induced mitochondrial damage and apoptosis in human endothelial cells are inhibited by vitamin C. Free Radic. Biol. Med..

[52] Shen X., Wang C., Liang N., Liu Z., Li X., Zhu Z.J., Merriman T.R., Dalbeth N., Terkeltaub R., Li C. (2021). Serum Metabolomics Identifies Dysregulated Pathways and Potential Metabolic Biomarkers for Hyperuricemia and Gout. Arthritis Rheumatol..

[53] Weidinger D., Jamal Jameel K., Alisch D., Jacobsen J., Bürger P., Ruhe M., Yusuf F., Rohde S., Störtkuhl K., Kaufmann P. (2022). OR2AT4 and OR1A2 counterregulate molecular pathophysiological processes of steroid-resistant inflammatory lung diseases in human alveolar macrophages. Mol. Med..

[54] Orecchioni M., Kobiyama K., Winkels H., Ghosheh Y., McArdle S., Mikulski Z., Kiosses W.B., Fan Z., Wen L., Jung Y. (2022). Olfactory receptor 2 in vascular macrophages drives atherosclerosis by NLRP3-dependent IL-1 production. Science.

[55] Nishida A., Miyamoto J., Shimizu H., Kimura I. (2021). Gut microbial short-chain fatty acids-mediated olfactory receptor 78 stimulation promotes anorexigenic gut hormone peptide YY secretion in mice. Biochem. Biophys. Res. Commun..

[56] Leask M.P., Sumpter N.A., Lupi A.S., Vazquez A.I., Reynolds R.J., Mount D.B., Merriman T.R. (2020). The Shared Genetic Basis of Hyperuricemia, Gout, and Kidney Function. Semin. Nephrol..

[57] Dalbeth N., Gosling A.L., Gaffo A., Abhishek A. (2016). Gout. Lancet.

[58] Liu Y.R., Wang J.Q., Li J. (2023). Role of NLRP3 in the pathogenesis and treatment of gout arthritis. Front. Immunol..

[59] Liu B., Zhao C., Wang J., Du S.M., Bu R.Z., Zheng X.Y., Zhou J., Wang J. (2023). Quality evaluation of Biqi Capsules based on HPLC fingerprint and multi-component analysis. Chin. Tradit. Herb. Drugs.

[60] Liu J.T., Zhang Y., Bu R.Z., Zhao H.P., Zhao Y., Zhang H.B., Xu J., Zhang T.J., Wang L., Liu C.X. (2021). Indentification of chemical components and blood components of Biqi Capsules by UPLC-Q/TOF-MS. Chin. Tradit. Herb. Drugs.

[61] Sekula P., Del Greco M.F., Pattaro C., Köttgen A. (2016). Mendelian Randomization as an Approach to Assess Causality Using Observational Data. J. Am. Soc. Nephrol..

[62] Emdin C.A., Khera A.V., Kathiresan S. (2017). Mendelian Randomization. JAMA.

[63] Zhang W., Ghosh D. (2021). A general approach to sensitivity analysis for Mendelian randomization. Stat. Biosci..

