# Supplementary material for: Exploring Therapeutic Potential of Bi-Qi Capsules in Treatment of Gout by Discovering Crucial Drug Targets
Source: Pharmaceuticals (Basel). 2025 Apr 24;18(5):618. doi: 10.3390/ph18050618 (PMC12114690; doi:10.3390/ph18050618)
Supplement: Supplementary file 1 [file pharmaceuticals-18-00618-s001.zip › pharmaceuticals-3487575-supplementary.pdf]

Supplementary Materials

Exploring therapeutic potential of Bi-Qi capsule in treatment of gout by discovering crucial drug targets

Jing Xie [1,2,3], Yu Zhang [2,3], Rong Ren [2,3], Ruizhen Bu [4], Liying Chen [3,5], Juezhuo Hou [2,3], DanDan Shang [2,3], Yadong Liu [2,3], Dan Wang [1,6], Tao Wang [1,6*], and Hong Zhou [2,3] *

1 State Key Laboratory of Bioactive Substance and Function of Natural Medicines, Institute of Materia Medica, Chinese Academy of Medical Sciences and Peking Union Medical College, Beijing 100050, China;
2 Tianjin pharmaceutical Da Ren Tang Group Co., Ltd, 17 Baidi Road, Nankai District, Tianjin 300193, China;
3 Tianjin Key Laboratory of Quality Control in Chinese Medicine, 21 10th Street, Binhai New Area, Tianjin 300457, China;
4 Tianjin Darentang Jingwanhong Pharmaceutical Co., Ltd., 20 Daming Road, Xiqing District, Tianjin 300112, China;
5 Tianjin Pharmaceutical Da Ren Tang Group Co., Ltd Traditional Chinese Pharmacy Research Institute, 21 10th Street, Binhai New Area, Tianjin 300457, China;
6 Tianjin University of Traditional Chinese Medicine, 10 Poyang Lake Road, Jinghai District, Tianjin 301617, China;

Correspondence:

Hong Zhou,

E-mail: tjzhouhong@163.com

Tao Wang,

E-mail: wangtao@tjutcm.edu.cn

Tables

Table S1 Data sources of the research

Table S2 MR analysis for causal relationship of crucial targets and gout.

Table S3 Results for heterogeneity test and horizontal pleiotropic test of crucial targets and gout

Table S4 MR analysis for causal relationship of PTPRS and HUA.

Table S5 Results for heterogeneity test and horizontal pleiotropic test of PTPRS and HUA.

Table S1 Data sources of the research

**Data Name**	**Sources**
Differentially expressed genes（DEGs)in diseases	GEO database(GSE160170)
Drug targets	Swiss Target Prediction、TCMSP、STITCH
Mendelian randomization analysis	GWAS Database(GCST90038687,GCST008972)
Single cell RNA sequencing,	CDCP、Cell Blast、DISCO,BioGPS Database、mRNALocater Database
Transcription factor TF, endogenous RNA	ChEA3 Database、Starbase Database
Molecular docking	RSCB PDB Database

Table S2 MR analysis for causal relationship of crucial targets and gout.

Exposure	Outcome	method	pval	OR
eqtl-a-ENSG00000130037（KCNA5）	GOUT	MR Egger	0.5708	0.9938
		Weighted median	0.0356	0.9956
		Inverse variance weighted	0.0039	0.9947
		Simple mode	0.2626	0.9956
		Weighted mode	0.2075	0.9959
eqtl-a-ENSG00000073756（PTGS2）		MR Egger	0.3270	1.0007
		Weighted median	0.1306	1.0008
		Inverse variance weighted	0.0313	1.0010
		Simple mode	0.1178	1.0015
		Weighted mode	0.1627	1.0008
eqtl-a-ENSG00000232810（TNF）		MR Egger	0.1035	0.9970
		Weighted median	0.0004	0.9972
		Inverse variance weighted	0.0002	0.9971
		Simple mode	0.1966	0.9971
		Weighted mode	0.0114	0.9972

Table S3 Results for heterogeneity test and horizontal pleiotropic test of crucial targets and gout

Exposure	Outcome	MR Egger_intercept	MR Egger pval	Cochrane's Q test	Cochrane's Q pval
eqtl-a-ENSG00000130037（KCNA5）	Gout	8.35E-05	0.9245	1.3036	0.5211
eqtl-a-ENSG00000073756（PTGS2）		8.71E-05	0.5595	4.0501	0.8816
eqtl-a-ENSG00000232810（TNF）		2.45E-05	0.9230	8.5483	0.2867

Table S4 MR analysis for causal relationship of PTPRS and HUA.

Exposure	Outcome	Method	pval	OR
eqtl-a-ENSG00000105426(PTPRS)	HUA	Inverse variance weighted	0.0477	1.0457
		MR Egger	0.9817	0.9988
		Weighted median	0.1424	1.0359
		Simple mode	0.3995	1.0404
		Weighted mode	0.3494	1.0296

Table S5 Results for heterogeneity test and horizontal pleiotropic test of PTPRS and HUA.

Exposure	Outcome	MR Egger_intercept	MR Egger pval	Cochrane's Q test	Cochrane's Q pval
eqtl-a-ENSG00000105426(PTPRS)	HUA	0.005372	0.4292	2.2862	0.3188

Figures

Figure S1 (A) The expression level of KCNA5, PTGS2 and TNF in normal and gout group. (B) Funnel plots of the MR analysis for three crucial targets on gout. (C) Leave-one-out (LOO) analysis of the MR analysis for sensitivity analyses of three crucial targets on gout.

Figure S2 Funnel plots of the MR analysis for PTPRS and HUA, and LOO of the MR analysis for sensitivity analyses of PTPRS and HUA.

Figure S3 HPLC(A) and MS (B) fingerprints of Bi-Qi Capsules

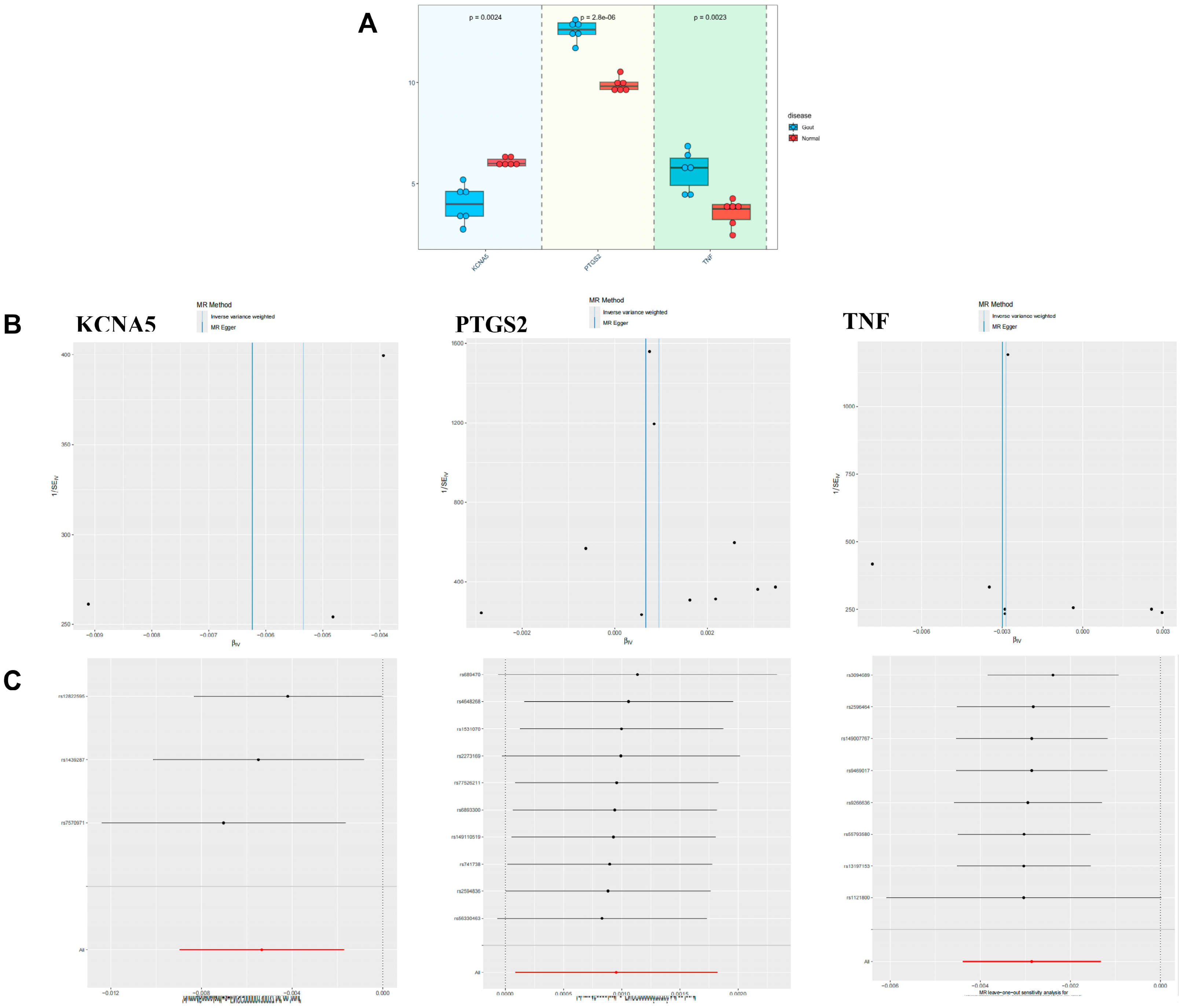

Figure S1 (A) The expression level of KCNA5, PTGS2 and TNF in normal and gout group. (B) Funnel plots of the MR analysis for three crucial targets on gout. (C) Leave-one-out (LOO) analysis of the MR analysis for sensitivity analyses of three crucial targets on gout.

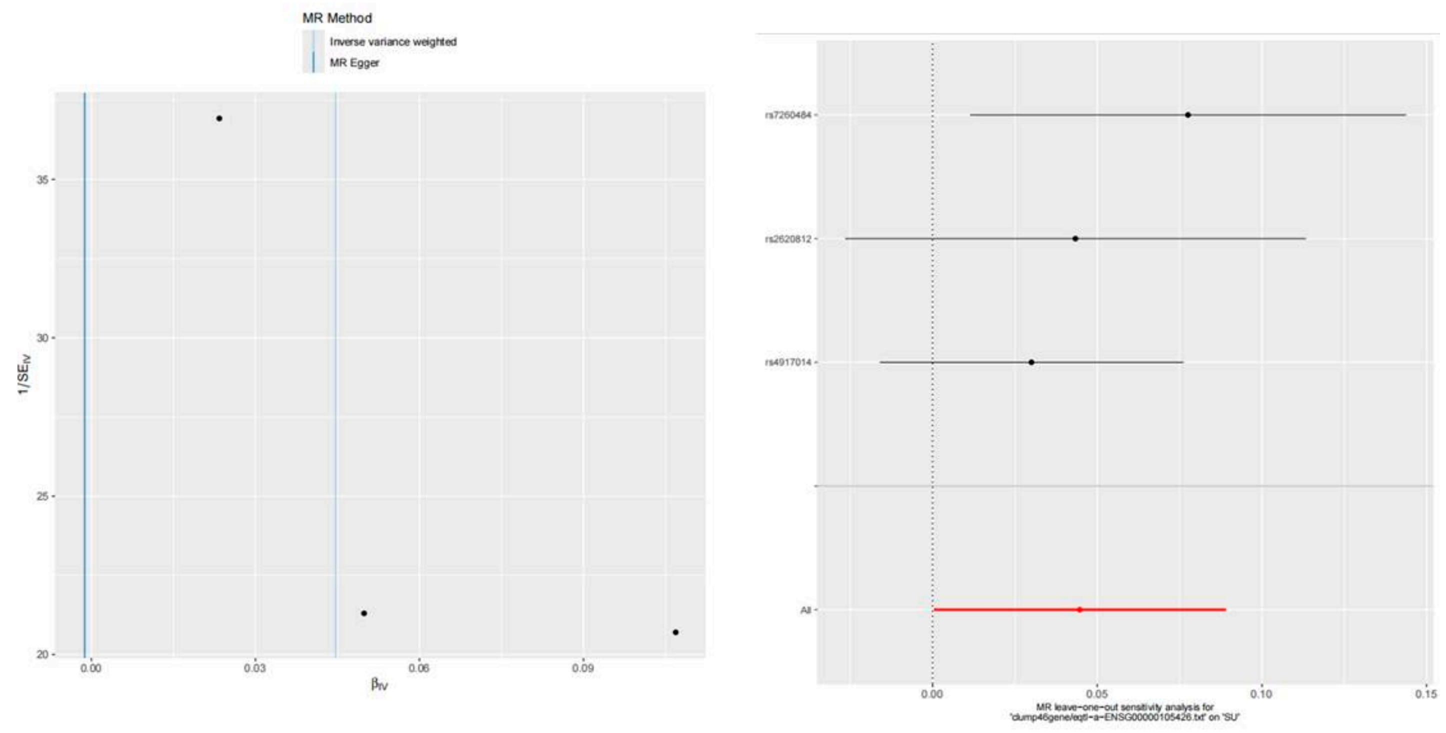

Figure S2 Funnel plots of the MR analysis for PTPRS and HUA, and LOO of the MR analysis for sensitivity analyses of PTPRS and HUA.

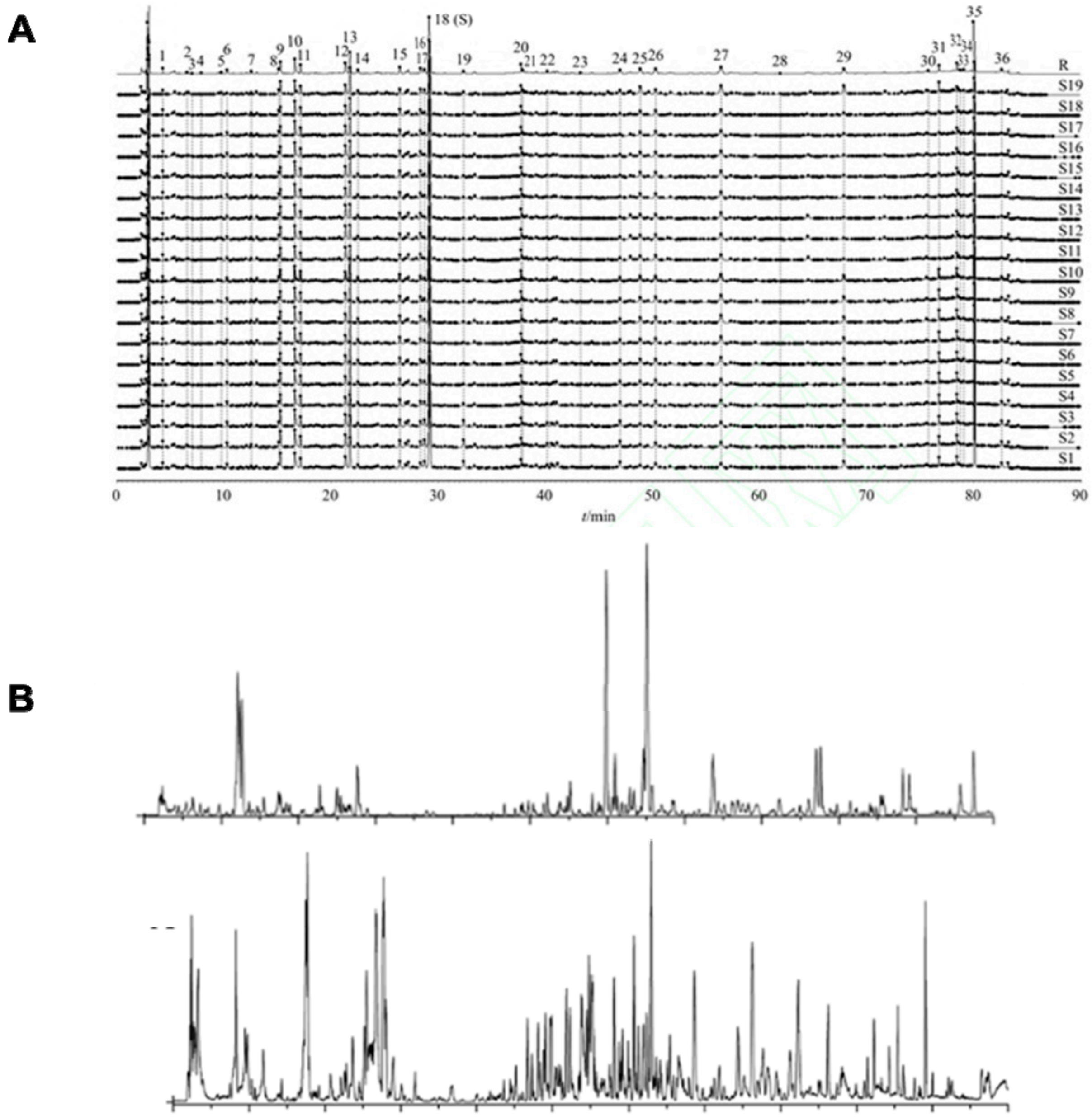

Figure S3 HPLC(A) and MS (B) fingerprints of Bi-Qi Capsules.

(A) Danshensu (peak 6), strychnine (peak 10), brucine (peak 11), liquiritin (peak 13), ferulic acid (peak 14), rosmarinic acid (peak 15), ginsenoside Rg1 (peak 16), salvianolic acid B (peak 18), ginsenoside Rb1(peak 20), ammonium glycyrrhizinate (peak 22), ligustilide (peak 27), tanshinone IIA(peak 30), pachymic acid (peak 33)
